# Causes of Maternal Mortality Decline in Matlab, Bangladesh

**DOI:** 10.3329/jhpn.v27i2.3325

**Published:** 2009-04

**Authors:** Mahbub Elahi Chowdhury, Anisuddin Ahmed, Nahid Kalim, Marge Koblinsky

**Affiliations:** ICDDR,B, GPO Box 128, Dhaka 1000, Bangladesh

**Keywords:** Causes of death, Delivery, Health services, Health facilities, Healthcare, Maternal health, Maternal mortality, Obstetric care, Risk factors, Bangladesh

## Abstract

Bangladesh is distinct among developing countries in achieving a low maternal mortality ratio (MMR) of 322 per 100,000 livebirths despite the very low use of skilled care at delivery (13% nationally). This variation has also been observed in Matlab, a rural area in Bangladesh, where longitudinal data on maternal mortality are available since the mid-1970s. The current study investigated the possible causes of the maternal mortality decline in Matlab. The study analyzed 769 maternal deaths and 215,779 pregnancy records from the Health and Demographic Surveillance System (HDSS) and other sources of safe motherhood data in the ICDDR,B and government service areas in Matlab during 1976-2005. The major interventions that took place in both the areas since the early 1980s were the family-planning programme plus safe menstrual regulation services and safe motherhood interventions (midwives for normal delivery in the ICDDR,B service area from the late 1980s and equal access to comprehensive emergency obstetric care [EmOC] in public facilities for women from both the areas). National programmes for social development and empowerment of women through education and microcredit programmes were implemented in both the areas. The quantitative findings were supplemented by a qualitative study by interviewing local community care providers for their change in practices for maternal healthcare over time. After the introduction of the safe motherhood programme, reduction in maternal mortality was higher in the ICDDR,B service area (68.6%) than in the government service area (50.4%) during 1986-1989 and 2001-2005. Reduction in the number of maternal deaths due to the fertility decline was higher in the government service area (30%) than in the ICDDR,B service area (23%) during 1979-2005. In each area, there has been substantial reduction in abortion-related mortality—86.7% and 78.3%—in the ICDDR,B and government service areas respectively. Education of women was a strong predictor of the maternal mortality decline in both the areas. Possible explanations for the maternal mortality decline in Matlab are: better access to comprehensive EmOC services, reduction in the total fertility rate, and improved education of women. To achieve the Millenium Development Goal 5 targets, policies that bring further improved comprehensive EmOC, strengthened family-planning services, and expanded education of females are essential.

## INTRODUCTION

Bangladesh is unique among developing countries as the safe motherhood process indicators, such as use of skilled birth attendants (SBAs) and caesarean section, are relatively low (13% and 3.5% respectively) (1) given the maternal mortality ratio (MMR) of 322 per 100,000 livebirths (2). Within Bangladesh, there is also a great variation—with some divisions achieving much higher levels of use of maternal healthcare services (skilled birthing care, caesarean-section rates) than others.

Such anomalies have also been observed in Matlab, the field site of ICDDR,B, located in some 55 km southeast of Dhaka, where longitudinal data are available. The Matlab area of ICDDR,B is divided into ICDDR,B and government service areas. The ICDDR,B service area has received extensive health and family-planning services since 1978 whereas the government service area receives routine government health services.

During 1976-2000, maternal mortality declined from 412 to 233 per 100,000 pregnancies in the ICDDR,B service area in Matlab (3). The use of skilled birthing care in the mid-1970s when obstetric mortality was first recorded was almost non-existent (2.1%) (4). In the late 1990s when use was still well below 30% of total births (5), maternal mortality had declined by nearly 43% (3). Caesarean-section rates increased in recent years (6.8% in 2005) (6) but remained low during 1990-2001 (0.2-2.7%) (4). While the SBAs may have played a significant role in early detection, management, or referral of complicated cases in recent years (skilled attendance at birth was 50% in 2005) (3), the reasons for the declining trend in maternal mortality in the ICDDR,B service area initiated in the mid-1970s remain largely unexplained by the process indicators.

The government service area—the comparison area—in Matlab also reports a decline in maternal mortality from 451 to 353 per 100,000 pregnancies during 1976-2000 (3). This reduction in mortality in the government service area was obtained with very low use of skilled care at birth provided through regular government services. In this area, the proportion of births with a health professional was only 3.7% in the late 1990s and below 10.0% until 2001 (4). Caesarean-section rates also remained low (0.1-1.1%) during 1990-2000 (4).

To explore the decline in the MMR in Matlab, we go beyond the safe motherhood programme elements to investigate the possibility of other factors contributing to the decline in the MMR using both quantitative and qualitative means. To explore reasons for the decline of maternal mortality in Matlab during 1976-2005, participants of a national stakeholders' meeting developed the following hypotheses that guided our research:

- Increased use of skilled care at birth
- Improved safer abortion practices
- Changes in causes of maternal mortality to those less amenable to management
- Societal changes (socioeconomic develop ment, education of women, empowerment of women)
- Improved birth-spacing
- Changes in fertility through the shift of risk-groups (high parity to lower parity; older age to younger age)
- Improved maternal nutritional status (role of improved maternal nutrition, e.g. body mass index and anaemia)

Among the above seven possible hypotheses, the last one has not been addressed in this paper due to non-availability of data; a brief overview of each of the other hypotheses is provided. This paper then addresses more in-depth research relating to the use of skilled care at birth using individual-level analysis, improved safer abortion practices using abortion ratios, changes in causes of mortality to those less amenable to management, changes in birth-spacing, and changes in fertility and their effects on the reduction in maternal mortality.

## MATERIALS AND METHODS

### Study site

The study used different secondary sources of data from Matlab where the Health and Demographic Surveillance System (HDSS) has systemically recorded demographic events, such as births, deaths, marriages, and migrations, of 220,000 people equally divided into two groups: one group under ICDDR,B service area and another under government service area since 1966.

The ICDDR,B service area has received extensive health and family-planning services since 1978 (7). Community health workers were deployed to visit women in their homes fortnightly to provide an integrated package of maternal and child healthcare by providing contraceptives, vaccines, oral rehydration solutions, vitamin A capsules, and nutrition education. Since the early 1980s, they also distributed safe delivery-kits and iron tablets to pregnant women. In 1987, a safe motherhood programme was piloted in half of the ICDDR,B service area (8). The programme was aimed at increasing the coverage of pregnancy, delivery, and postnatal care in the home by posting midwives in the communities. A basic emergency obstetric care (EmOC) facility was established in Matlab town. Women who had complications were provided with free transportation to the basic EmOC facility in Matlab or a referral hospital in the district headquarters when necessary. In 1990, the programme was expanded to cover the entire ICDDR,B service area. In 1996, the programme was redesigned for facility-based birthing, and during 1996-2001, the home-based birthing strategy gradually shifted to the facility-based strategy (5). All services in the ICDDR,B area were provided free of charge.

The government service area has offered regular government family-planning services since the late 1970s. Women in the government service area did not have access to safe motherhood services offered by ICDDR,B. However, from both ICDDR,B and government service areas, the distance to referral facilities at district headquarters was similar; the same referral facilities are used by women in both ICDDR,B and government service areas.

Details of services provided in the ICDDR,B service area have been described elsewhere (8). Table 1 shows the type and timing of different interventions in the ICDDR,B and government service areas in Matlab.

**Table 1.** Type and timing of interventions in Matlab ICDDR,B and government service areas, 1976-2005 (3)

Interventions	ICDDR,B service area	Government service area
Contraceptives	Introduced in 1978 by ICDDR,B, intensified over time. The total fertility rate declined from 4.5 in 1978 to 2.7 in 2005	Introduced in the late 1970s by the Government, intensified over time. The total fertility rate declined from 5.5 in 1978 to 2.8 in 2005
Menstrual regulation	Introduced in the late 1970s by government service providers	Introduced in the late 1970s by government service providers
Antenatal screening	Simple screening tool introduced in 1982 (community health workers), continues today	Not available
Training of TBAs	ICDDR,B trained TBAs during 1982-1999. The programme stopped in 2000	The Government trained TBAs during 1982-1999. The programme stopped in 2000
Access to skilled attendants	Four midwives posted in half of the area in 1987, expanded to full area in 1990, continues today with 8 midwives. During 1987-2005, the proportion of births with a skilled attendant increased from 5.0% to 53.0%	No midwives are practising in the area. During 1987-2005, the percentage of births with a skilled attendant increased from 2.3% to 14.2%
Access to comprehensive emergency obstetric care (EmOC)	Most women go to Chandpur town where there were two comprehensive EmOC hospitals during 1976-1990. The number of private clinics offering com prehensive EmOC increased dramatically in the 1990s. From 1987 onwards, ICDDR,B offered free transport for women to go to Chandpur. The caesarean rate increased from 0.2% in 1990 to 6.8% in 2005. During 1990-2001, the percentage of births with a caesarean section to save the mother's life rose from 0.3% to 0.9%	Most women go to Chandpur town where there were two comprehensive EmOC hospitals during 1976-1990. The number of private clinics offering comprehensive EmOC increased dramatically in the 1990s. The caesarean rate increased from 0.1% in 1990 to 4.2% in 2005. During 1990-2001, the percentage of births with a caesarean section to save the mother's life rose from 0.1% to 0.3%
Availability of antibiotics	Widely available in villages since the mid-1980s	Widely available in villages since the mid-1980s
Microcredit programmes	Introduced in the mid-1980s, ongoing	Introduced in the mid-1980s, ongoing
Education of women	The proportion of pregnant women without formal schooling decreased from 69% in 1976-1980 to 27% in 2001-2005	The proportion of pregnant women without formal schooling decreased from 73% in 1976-1980 to 28% in 2001-2005
Socioeconomic development	The major changes over the full period. The proportion of pregnant women classified as poor decreased from 31% in 1976-1980 to 1% in 2001-2005	The major changes over the full period. The proportion of pregnant women classified as poor decreased from 34% in 1976-1980 to 1% in 2001-2005

EmOC=Emergency obstetric care; MR=Menstrual regulation; TBAs=Traditional birth attendants

Reprinted (with minor modifications) from *The Lancet* (V. 370, 2007:1321). Chowdhury ME, Bot lero R, Koblinsky M, Saha SK, Dieltiens G, Ronsmans C. Determinants of reduction in maternal mortality in Matlab, Bangladesh: a 30-year cohort study, with permission from Elsevier

### Sources of data

Different secondary sources of data in Matlab were used for the study. The HDSS data were linked to several other sources of safe motherhood and socioeconomic data in Matlab, including pregnancy-monitoring cards, facility records for pregnancy and delivery care at the Matlab Hospital and subcentres, verbal autopsies, the Matlab Health and Socioeconomic Survey (1996), and periodical socioeconomic censuses (1982, 1996, and 2005). Verbal autopsies conducted during 1976-2005 under the HDSS, along with special maternal death reviews to validate maternal deaths, were used for determining the causes of maternal mortality (9-11). The study identified 215,779 pregnancy-records and 769 maternal deaths occurring in the study areas during 1976-2005.

In both ICDDR,B and government service areas, periodical socioeconomic census data on consumption of durable goods, e.g. table, chair, watch, television, and bicycle; housing facilities, e.g. types of toilet and sources of drinking-water; housing materials, e.g. types of wall and ownership of land, were collected. These data were used for determining the socioeconomic status of women/households by computing the asset quintile. To perform analysis, all the above sources of data were linked using the unique identification number of each person in the HDSS. Both descriptive and analytical analyses were performed.

In addition to the above secondary sources of data, findings of the study were also supplemented by qualitative research implemented in Matlab. Qualitative research methods included in-depth interviews and focus-group discussions with traditional birth attendants (TBAs) and village doctors (not medically trained but who often provide drugs from local shops and through home-visits). The objective of the qualitative study was to understand the possible changes in birth practices in the community by TBAs and village doctors over time.

### Definition of outcome and explanatory variables

A maternal death was defined as death of a woman while pregnant or within 90 days of termination of pregnancy, irrespective of duration of pregnancy or termination method, excluding deaths from intentional and unintentional injuries. Deaths were further classified into direct obstetric causes (including antepartum, intrapartum and postpartum haemorrhage, hypertensive disorder of pregnancy, dystocia, and sepsis), abortion, and indirect causes. We pooled all spontaneous and induced abortion-related deaths into one group.

Skilled care at birth was defined as care from a medically-trained provider if the trained provider was present at any time during labour, delivery, or the immediate postpartum period in the home or in the facility. Women were classified as having received basic EmOC from a trained provider (doctor, nurse, midwife, and paramedic) if the trained care provider was present at any time during labour, delivery, or the immediate postpartum period, whether or not they actually conducted the birth. Births taking place in higher-level facilities where caesarean section and blood transfusion were available outside the Matlab area were classified as comprehensive EmOC. Women first seen by an ICDDR,B midwife in the home and then referred to a higher-level facility outside the Matlab area were categorized as referred through the ICDDR,B system. Birth-spacing was defined as birth-to-pregnancy intervals measured as years between the previous pregnancy outcome and the conception of the next pregnancy.

## RESULTS

### Hypothesis 1: Increased use of skilled care at birth

In the ICDDR,B service area, after the introduction of the safe motherhood intervention, the use of skilled care at delivery increased from 7.3% during 1987-1989 to 39.5% during 2001-2005. During the same period, maternal mortality declined from 417 to 131 per 100,000 pregnancies (68.6% reduction) (Table 2). In the government service area, there was a 50.4% reduction in maternal mortality during the same period despite the very low use of skilled care (14.2% in 2005) (6). In the early 1990s, the caesarean-section rate was well below 1% in both the areas and increased to 6.8% and 4.2% in the ICDDR,B and government service areas respectively by 2005.

**Table 2.** Trends in maternal deaths during 1976-2005 in Matlab, pre- and post-introduction of the safe motherhood intervention in ICDDR,B service area

Time period	ICDDR,B service area		Government service area	
	No. of pregnancies	Maternal deaths per 100,000 pregnancies	No. of pregnancies	Maternal deaths per 100,000 pregnancies
Before safe motherhood intervention				
1976-1980	18,919	412.3(78)	21,042	451.5 (95)
1981-1985	18,113	414.1 (75)	22,170	505.2 (112)
1986-1989	14,131	417.5 (59)	17,112	414.9 (71)
After safe motherhood intervention								
1990-1995	18,126	281.4 (51)	21,832	361.9 (79)
1996-2000	14,573	233.3 (34)	16,716	353.0 (59)
2001-2005	16,035	131.0 (21)	17,010	205.8 (35)

Figures in parenthesis indicate the number of deaths

Chowdhury *et al*. analyzed the Matlab safe motherhood data during 1976-2005 to gain insights into the role of the safe motherhood programme in reducing maternal mortality by examining the trends in the two areas (3). The study found that the speed of the decline in maternal mortality after 1989, following the introduction of the safe motherhood programme, was faster in the ICDDR,B service area than in the government service area, although the difference was not statistically significant (p=0.09). While the authors could not fully explain the role of skilled birth attendants (SBAs) in reduction of maternal mortality in Matlab, they concluded that increased access to comprehensive EmOC might have played a vital contribution to the reduction of maternal mortality.

To further explore the relationship between the use of skilled care at birth and maternal mortality, we did more in-depth analysis of data on safe motherhood in the ICDDR,B service area in Matlab during 1987-2005.

Individual-level analysis linking the birth-records, including maternal deaths, with the use and non-use of data on skilled care around the time of labour and delivery using the data on safe motherhood from the ICDDR,B service area only during 1987-2005 showed that 22.8% (n=13,507) of 59,165 pregnancies received care from trained providers.

Seeking care from trained providers at birth in the ICDDR,B service area increased dramatically over time—from 4.9% in 1987 to 50.9% in 2005. Overall, 5.0% (n=2,925) of the pregnant women (n=59,165) sought care from the comprehensive EmOC facilities. Birth in the comprehensive EmOC facilities increased considerably—from 0.5% in 1987 to 12.6% in 2005. About 55% (n=1,596) of the women who sought care from comprehensive EmOC facilities bypassed the Matlab safe motherhood programme, and the remaining 45% (n=1,329) were referred through the programme. The proportion of women who bypassed the programme for seeking comprehensive EmOC consistently increased from 1.1% to 9.0% during 1990-2005 respectively.

In the ICDDR,B service area, there were 173 maternal deaths during 1987-2005, resulting in maternal mortality of 292 per 100,000 pregnancies. Although the overall use of skilled care at birth was low among the women who died, the majority (57.8%) came in contact with skilled care providers (basic EmOC–20.8% and comprehensive EmOC–37.0%) at some point during the birthing process.

During 1987-2005, there was a huge reduction in maternal mortality in the ICDDR,B service area. Compared to 1987-1991, maternal mortality was 59% lower in 2002-2005 [crude odds ratio (OR)=0.41, 95% confidence interval (CI) 0.25-0.67]. However, as a whole, seeking skilled care was strongly associated with increased maternal mortality. Women who sought comprehensive EmOC and basic EmOC were about 57 and 6 times more likely to die respectively compared to those who did not seek care from trained providers or any professional care around the time of delivery (Table 3). However, the association between maternal mortality and care-seeking around labour and delivery differed by year of birth of the baby. The interaction between care-seeking and year of birth was significant (p<0.01). During 1987-2005, the reduction in maternal mortality due to use of comprehensive EmOC was 26% per year. The corresponding reduction for basic EmOC and referral was 8% per year. Maternal mortality also declined at an annual rate of 8% among non-users of skilled care.

**Table 3.** Maternal mortality in ICDDR,B service area by year of birth and the highest level of care at time of labour and delivery, 1987-2005

Year of birth and level of care	Maternal mortality		
	Maternal deaths per 100,000 pregnancies (no. of deaths)	Crude odds ratio (95% confidence interval)	Adjusted odds ratio (95% confidence interval)
Year of birth			
1987-1991	394 (66)	1.00	1.00
1992-1996	365 (53)	0.93 (0.64-1.33)	0.84 (0.57-1.23)
1997-2001	219 (33)	0.55 (0.36-0.84)	0.51 (0.32-0.81)
2002-2005	163 (21)	0.41 (0.25-0.67)	0.22 (0.12-0.40)
Highest level of care at time of labour and delivery			
No professional care	160 (73)	1.00	1.00
Basic EmOC and referral	521 (62)	3.30 (2.32-4.60)	6.02 (4.23-8.60)
Comprehensive EmOC	2,382 (38)	15.24 (10.26-22.63)	56.63 (36.22-88.53)

EmOC=Emergency obstetric care

During the early years of the programme, when a few women sought professional care around the time of labour and delivery, mortality among those seeking care directly from a comprehensive EmOC facility (18,811 per 100,000 pregnancies) was extremely high which gradually declined as more women sought care in these facilities (Table 4).

**Table 4.** Maternal mortality ratios by types of care received among women in ICDDR,B service area in Matlab, 1987-2005

Type of care	Mortality per 100,000 pregnancies by type of care received			
	1987-1991	1992-1996	1997-2001	2002-2005
Highest level of care at time of labour and delivery								
No professional care	206.9 (31)	219.1 (26)	96.2 (11)	67.8 (5)
Basic EmOC and referral	967.4 (16)	693.0 (17)	512.2 (17)	267.6 (12)
Comprehensive EmOC only	18,811.9 (19)	5,434.8 (10)	1,597.4 (05)	401.2 (4)
Any care	394.4 (66)	365.4 (53)	219.1 (33)	163.3 (21)

Figures in parenthesis indicate the number of deaths; EmOC=Emergency obstetric care

In the later years (2002-2005) of the programme, although overall mortality was still high (163 per 100,000 pregnancies), women giving birth in the home without a trained care provider had low levels of maternal mortality (68 per 100,000 pregnancies).

### Hypothesis 2: Improved safer abortion practices

There was substantial reduction in abortion-related mortality in both ICDDR,B and government service areas. During 1976-2005, there were 143 abortion-related deaths in both the areas, resulting in an overall mortality of 66.3 per 100,000 pregnancies.

After the introduction of safe menstrual regulation (MR) services in the primary healthcare settings in the early 1980s, abortion-related mortality declined from 93.9 in 1981-1985 to 12.5 in 2001-2005 per 100,000 pregnancies, i.e. 86.7% reduction, in the ICDDR,B service area (Table 5). The corresponding reduction in the government service area was from 108.3 to 23.5 per 100,000 pregnancies, i.e.78.3% reduction.

**Table 5.** Trends in abortion-related deaths in Matlab during 1976-2005

Annual trend in deaths	ICDDR,B service area		Government service area	
	No. of pregnancies	Abortion-related deaths/100,000 pregnancies	No. of pregnancies	Abortion-related deaths/100,000 pregnancies
1976-1980	18,919	100.4 (19)	21,042	52.3 (11)
1981-1985	18,113	93.9 (17)	22,170	108.3 (24)
1986-1989	14,131	63.7 (9)	17,112	105.2 (18)
1990-1995	18,126	55.2 (10)	21,832	68.7 (15)
1996-2000	14,573	27.4 (4)	16,716	59.8 (10)
2001-2005	16,035	12.5 (2)	17,010	23.5 (4)

Figures in parenthesis indicate the number of deaths

### Hypothesis 3: Changes in causes of maternal mortality to those less amenable to management

**Fig. 1.**
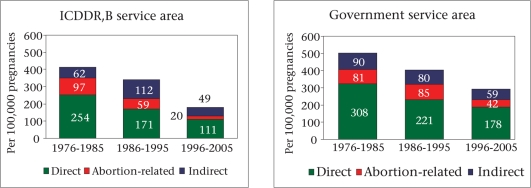
Trend in maternal death by causes in ICDDR,B and government service areas during 1976-2005 in Matlab

Figure 1 shows the trends in maternal mortality by cause and area. During 1976-2005, there has been a substantial reduction in direct obstetric mortality (not including abortion-related mortality) in both the areas. In the ICDDR,B service area, direct obstetric mortality declined from 254 per 100,000 pregnancies during 1976-1985 to 111 per 100,000 pregnancies during 1996-2005 while the corresponding decline in the government service area was from 308 to 178 per 100,000 pregnancies. As discussed above, there has also been a significant decline in abortion-related mortality. In both the areas, during 1976-2005, there has been no change in death rates due to indirect causes. Thus, there has been a shift in the pattern of deaths towards those causes less amenable to management.

**Fig. 2.**
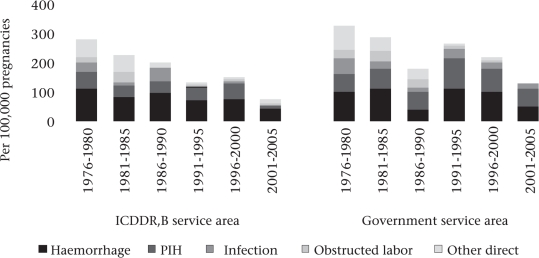
Trends in direct obstetric mortality in ICDDR,B and government service areas by cause in Matlab 1976-2005

**Fig. 3.**
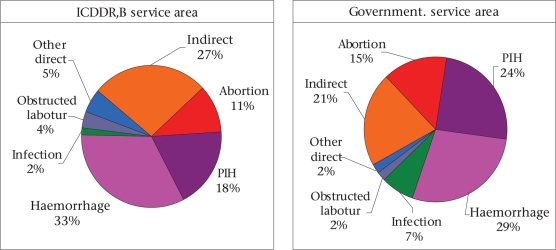
Causes of maternal deaths in Matlab by area during 1996-2005

During 1976-2005, there has been substantial reduction in deaths due to postpartum haemorrhage, pregnancy-induced hypertension (PIH), infection, obstructed labour, and other direct causes in the ICDDR,B service area whereas, in the government service area, the reduction occurred in haemorrhage, infection, and obstructed labour (Fig. 2). Haemorrhage was and continues to be the leading cause of maternal death in both the study areas, although there was substantial reduction in its death rate. In the ICDDR,B service area, deaths due to haemorrhage decreased from 111 in 1976-1980 to 44 in 2001-2005 per 100,000 pregnancies while, in the government service area, the corresponding reduction was from 100 to 53 per 100,000 pregnancies. Proportionate causes of maternal deaths were similar in both ICDDR,B and government service areas during 1996-2005 (Fig. 3). Haemorrhage, PIH, and abortion were the three major causes of direct obstetric mortality in both the areas. Death due to infection was low in each area (2% in ICDDR,B service area and 7% in government service area). About a quarter of maternal deaths were due to indirect causes in both the areas.

PIH=Pregnancy-induced hypertension

PIH=Pregnancy-induced hypertension

The ICD-10 definition of maternal mortality excludes deaths from accidents and injuries. However, 20% of deaths of pregnant unmarried women in Matlab were due to suicide compared to 5% of deaths of married women, and pregnant female adolescents were nearly three times more likely to die due to violence compared to non-pregnant girls (12,13).

### Hypothesis 4: Societal changes (socioeconomic changes, empowerment of women, female education)

There have been major sociodemographic changes during 1976-2005 in Matlab in both the areas (Table 6). The proportion of pregnancies among women with no formal education in Matlab decreased by about half—from 68.2% during 1976-1985 to 35.8% during 1996-2005. During 2001-2005, more than half of the pregnant women had five or more years of schooling. Our analysis of change in the proportion of pregnancies by the asset quintile (based on a fixed list of assets over time) showed that there has been major socioeconomic improvement in Matlab during the study period. The proportion of pregnant women living in the highest asset quintile increased from 5.1% during 1976-1985 to 41.6% during 1996-2005. On the other hand, the proportion of pregnant women in the lowest asset quintile decreased from 30.6% during 1976-1985 to 2.1% during 1996-2005.

**Table 6.** Change in sociodemographic characteristics of pregnant women in Matlab during 1976-2005

Sociodemographic characteristics	% of pregnant women			
	1976-1985	1986-1995	1996-2005	Total
	(n=80,244)	(n=71,201)	(n=64,334)	(n=215,779)
Completed years of schooling				
0	68.2	59.3	35.8	55.6
1-4	12.1	11.6	12.5	12.1
5-7	13.6	16.8	25.5	18.2
≥8	2.9	7.2	24.4	10.8
Unknown	3.2	5.1	1.8	3.4
Household asset quintile				
Poorest	30.6	19.7	2.1	18.5
Poorer	25.1	22.8	9.5	19.7
Poor	20.1	18.2	14.1	17.7
Less	15.9	18.6	21.9	18.6
Least	5.1	13.0	41.6	18.6
Unknown	3.3	7.7	10.8	7.0
Pregnancy order				
1	21.1	22.1	27.8	23.4
2-3	31.5	34.4	40.5	35.1
4-5	21.5	22.4	20.3	21.4
≥6	25.9	21.2	11.5	20.1
Maternal age (years)				
≤19	20.9	12.2	11.1	15.1
20-29	50.7	62.3	57.3	56.5
30-39	25.0	22.4	28.9	25.3
≥40	3.4	3.1	2.7	3.1

The demographic composition of births also changed dramatically: pregnancies of order six or more decreased from 25.9% during 1976-1985 to 11.5% during 1996-2005, and the proportion of first pregnancies increased from 21.1% to 27.8%. Pregnancies in women aged less than 20 years decreased from 20.9% during 1976-1985 to 11.1% during 1996-2005.

There has been substantial reduction over time in the number of maternal deaths among women with more education in Matlab (Table 7). During 1976-1985 and 1996-2005, mortality declined by 36.7% among women with pregnancies who had no formal education. The corresponding decline in deaths among women with at least eight years of schooling was 66.2%. Chowdhury *et al*., in analysis of the determinants of maternal mortality, found that education of women was a very strong predictor of survival. Result of the study showed that maternal mortality was about three times lower among women with eight or more years of education compared to women without any formal education (adjusted OR=0.36, 95% CI 0.24-0.53) (3).

**Table 7.** Variation in maternal death by maternal education and household asset quintile, Matlab, 1976-2005

Socioeonomic characteris	1976-1985		1986-1995		1996-2005		1976-1985 and 1996-2005 % declined
	No.	Maternal deaths/100,000 pregnancies	No.	Maternal deaths/100,000 pregnancies	No.	Maternal deaths/100,000 pregnancies	
Completed years of schooling							
0	54,712	515.4	42,229	435.7	23,008	326.0	36.7
1-4	9,739	328.6	8,282	362.2	8,051	385.0	-17.1
5-7	10,882	312.4	11,944	259.5	16,428	146.1	53.2
≥8	2,358	339.3	5,144	116.6	15,718	114.5	66.2
Unknown	2,553	156.7	3,602	249.9	1,129	88.6	-
household asset quintile								
Poorest	24,516	489.5	14,014	413.9	1,337	224.4	54.1
Poorer	20,099	457.7	16,254	375.3	6,107	327.5	28.4
Poor	16,139	458.5	12,972	462.5	9,059	242.9	47.0
Less poor	12,753	376.4	13,236	309.8	14,117	255.0	32.2
Least poor	4,108	389.5	9,227	238.4	26,781	190.4	51.1
Unknown	2,629	380.4	5,498	327.4	6,933	245.2	-

On the other hand, there was no specific pattern for change in reduction in maternal mortality over time among the asset quintiles. In their analysis, Chowdhury *et al*. found a considerable poor-rich gap in maternal mortality: the crude OR comparing women in the richest and the poorest quintile was 0.49 (95% CI 0.38-0.63) but the differentials by the asset quintile disappeared after adjusting for other covariates (3).

### Hypothesis 5: Change in birth-spacing

**Fig. 4.**
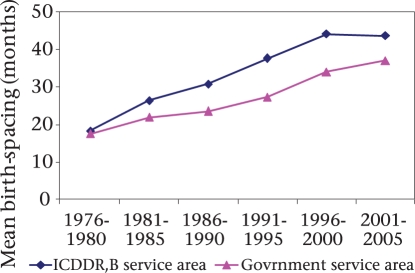
Mean birth-spacing (months) for birth-to-pregnancy interval of mother over time in Matlab, Bangladesh

To investigate the effect of change in birth-spacing on reduction in maternal mortality, we examined the change in risk of maternal deaths for change in birth-to-pregnancy intervals. When the date of conception was not recorded, it was derived by subtracting the last menstrual period (LMP) date from the previous date of birth. If the LMP date was unknown, a duration of 280 days was assumed for livebirths, 252 days for stillbirths, and 84 days for abortions as considered by another study (14). In total, 215,680 birth-records and 769 maternal deaths from both ICDDR,B and government service areas occurring during 1976-2005 were included in this analysis. During 1976-2005, the mean birth-spacing interval increased in both the areas but the increment in the ICDDR,B service area was significantly higher compared to the government service area (p<0.05) (Fig. 4). In the ICDDR,B service area, the mean birth-spacing interval increased from 18.3 to 43.7 months during 1976-1980 and 2001-2005. The corresponding increase in the government service area was 17.5 to 37.1 months. There was a lack of association between maternal mortality and birth-spacing (Table 8). The risk of mortality was the lowest for 1-2 year(s) of birth-to-pregnancy interval (210.4 per 100,000 pregnancies). The corresponding risk was slightly higher for ≤1 year (243.3 per 100,000 pregnancies) and >4 years (283.4 per 100,000 pregnancies). Overall, there was no association between risk of maternal mortality and birth-to-pregnancy intervals (p>0.05). After adjustment with other potential factors, neither short nor long birth-spacing was a risk factor for maternal mortality. Among women who had ≤1 year birth-to-pregnancy interval, the likelihood of maternal death was 1.2 times higher compared to those with 1-2 year(s) of spacing over the period of time (adjusted OR=1.24, 95% CI 0.89-1.70). The strength of the relationship was not stronger for those who had over four years of birth-to-pregnancy interval compared to the above group (adjusted OR=1.41, 95% CI 1.00-1.97).

**Table 8.** Odds ratios of maternal deaths for birth–to-pregnancy interval during 1976-2005 in Matlab, Bangladesh

Birth-spacing	Maternal deaths/100,000 pregnancies	Crude OR (95% CI)	Aadjusted OR∗ (95% CI)
First pregnancy (years)	477.4	2.30 (1.78-2.90)	3.23 (2.46-4.24)
≤1	243.3	1.20 (0.84-1.60)	1.24 (0.89-1.70)
1-2	210.4	1.0	1.0
2-3	235.3	1.12 (0.82-1.52)	1.14 (0.83-1.55)
3-4	228.1	1.10 (0.74-1.60)	1.20 (0.80-1.72)
>4	283.4	1.35 (0.97-1.90)	1.41 (1.00-1.97)
Unknown	792.9	3.80 (2.95-4.90)	3.80 (2.89-4.90)

∗Adjusted for the study areas, maternal age; CI=Confidence interval; OR=Odds ratio

### Hypothesis 6: Changes in fertility have impacted the MMR through the shift of risk-groups (high parity to lower parity; older age to younger age)

Women face obstetric risk of death everytime they are pregnant. One measure of fertility is the total fertility rate (TFR) which shows the average number of livebirths a woman is likely to have in her reproductive life. Bangladesh is considered a model country in reducing fertility. During 1979-2005, total fertility per woman aged 15-49 years declined in the government service area from 6.9 to 2.8 (6). The corresponding reduction in the TFR in the ICDDR,B service area was 4.9 to 2.7. Although the differences in availability of contraceptive service between the ICDDR,B service area and the government service area resulted in significantly lower fertility rates in the ICDDR,B service area before 2000, the fertility rates of the two areas have converged to around 3.

**Fig. 5.**
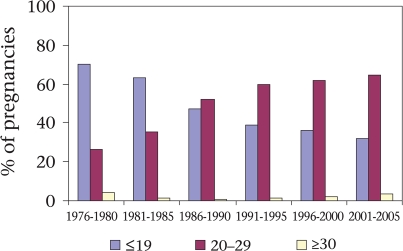
Distribution of maternal age (years) at first-order pregnancy during 1976-2005

As the TFR declined over the time period, the percentage of first births increased (overall 19% to 31% during 1976-2005) but, during 1976-1980, about 70% of the women had their first pregnancy within 19 years of age; this has consistently reduced to 32% during 2001-2005 (Fig. 5). We can assume that the higher age of women at first birth may have impacted on maternal mortality at the national level as well.

**Fig. 6.**
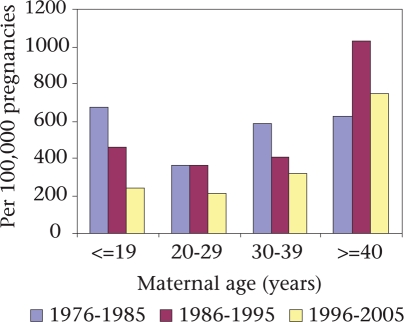
Trend in maternal deaths by maternal age, Matlab, 1976-2005

Paralleling the decline in fertility, the age at first births in the Matlab intervention area shifted from the higher-risk ≤19-year old women to the lower-risk 20-29 years age-group. Maternal deaths in both the age-groups have decreased over time but more so for the ≤19-year age-group. During 1976-1985, the mortality ratio among women of ≤19 years old was 659 per 100,000 pregnancies; this rate declined to 237 per 100,000 pregnancies during 1996-2005, i.e. a 64% decline. For women within the 20-29 years age-group, the corresponding reduction was only 42% giving a mortality ratio for this group of 355 per 100,000 pregnancies during 1976-1985 to 207 per 100,000 pregnancies during 1996-2005. Annual rates of the decline in maternal mortality for the ≤19 years and the 20-29 years age-group were 4% and 2% respectively during 1976-2005, both of which were statistically significant (p<0.05) (Fig. 6).

**Fig. 7.**
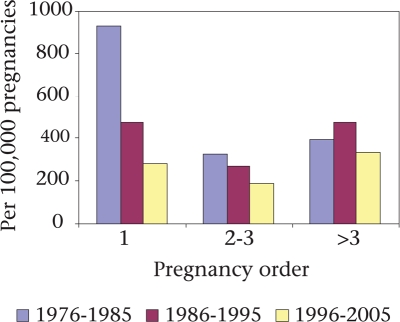
Trend in maternal deaths by pregnancy order, Matlab, 1976-2005

Also, there has been reduction in maternal deaths among those with pregnancy order 1-3. For the first-order pregnancy, the mortality rate of 924 per 100,000 pregnancies during 1976-1985 declined to 280 per 100,000 pregnancies during 1995-2005, i.e. a 70% decline. For the second- and third-order pregnancies, the corresponding reduction was 42% (Fig. 7). Annual rates of the decline in maternal mortality for the first-order and the second- and third-order pregnancy were 6% and 3% respectively, both of which were statistically significant (p<0.05). However, for higher than the third-order pregnancy, there was no apparent trend towards a change in mortality during 1976-2005.

**Fig. 8.**
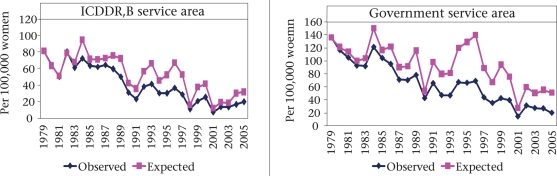
Effect of fertility on reduction in maternal deaths by areas, 1979-2005

Along with the TFR, there has been reduction in pregnancy rates in both ICDDR,B and government service areas. During 1979-2005, the pregnancy rate declined in the ICDDR,B service area from 5.5 to 3.2 pregnancies per woman (10-50 years of age). The corres ponding reduction in the government service area was 7.6 to 3.2 per woman. Assuming an age-specific constant pregnancy rate since 1979, expected maternal mortality in 2005 in the ICDDR,B and government service areas was estimated as 31.4 and 50.9 per 100,000 women respectively. The corresponding observed estimates of maternal deaths in the respective areas were 19.3 and 19.8 per 100,000 women. The overall declines in maternal mortality attributable to change in pregnancy rates were 23% and 30% respectively (Fig. 8).

A more realistic assessment of risk that takes into account both probability of becoming pregnant and probability of dying as a result of pregnancy-related complications accumulated across a woman's reproductive years is the lifetime risk of maternal death. In the ICDDR,B service area, one in every 41 women of reproductive age died during 1976-1980 due to pregnancy-related complication in her lifetime (Table 9). The corresponding figure in the government service area was one in 24 women.

**Table 9.** Trends in lifetime risk of maternal mortality by area, Matlab, 1978-2005

Year	Estimated MMR (per 100,000 livebirths)	Total fertility rate	LTR of maternal deaths (as odds)∗
ICDDR,B service area			
1978-1980	452.20	4.75	1 in 41
1981-1985	457.18	4.48	1 in 43
1986-1990	425.42	3.77	1 in 55
1991-1995	321.38	2.97	1 in 92
1996-2000	258.10	2.86	1 in 119
2001-2005	150.73	2.99	1 in 195
Government service area			
1978-1980	579.43	6.27	1 in 24
1981-1985	568.15	5.85	1 in 26
1986-1990	427.78	5.22	1 in 39
1991-1995	450.80	3.89	1 in 49
1996-2000	407.37	3.48	1 in 61
2001-2005	247.37	3.18	1 in 109

∗ LTR=1-(1-MMR)^PW∗TFR^, where MMR is expressed as a decimal. Pregnancy wastages of 14% and 17% in the ICDDR,B and the government service area were considered to adjust for pregnancies not ending in livebirths; LTR=Lifetime risk; MMR=Maternal mortality ratio; PW=Pregnancy wastage; TFR=Total fertility rate

During 1976-2005, the rate of reduction in lifetime risk in the government service area was faster than that in the ICDDR,B service area. In relative terms, this represents 79% and 78% reduction in lifetime risk of maternal mortality in the ICDDR,B and government service areas respectively. The more pronounced fertility effect on the decline of mortality in the government service area is related to the faster decline in fertility in that area.

## DISCUSSION

No single process indicator fully explains the declining trends in maternal mortality in Matlab. The resulting lowered maternal mortality rates in both ICDDR,B and government service areas are rather the result of multiple factors that differ in their contribution in each area.

Comprehensive EmOC contributed to reduction in the number of maternal deaths in both ICDDR,B and government service areas. Facilities for comprehensive EmOC are the same for women from both ICDDR,B and government service areas and located at a similar distance (the closest is about 45 minutes). During 1976-1986, the decline witnessed in the ICDDR,B service area occurred with almost no access to obstetric care. Since then, there has been an increase in use of comprehensive EmOC in the ICDDR,B service area in Matlab which has made a significant contribution to reduction in the number of maternal deaths. During 1987-1991, the use of skilled attendance was low (10.5%); women seeking comprehensive EmOC were most likely at a high risk, and mortality was high (18,811 per 100,000 pregnancies). During 2002-2005, more women sought skilled care at birth (40.5%), and mortality among those seeking comprehensive EmOC was lower but still relatively high at 401 per 100,000 pregnancies. This suggests that, in the early years, women who used comprehensive EmOC services arrived late with complications when skilled care could not save them. Another possible explanation is that hospital staff could not save these women due to poor quality of care. As time went on, women arrived for comprehensive EmOC in greater numbers; hence, women with more normal births were most likely among them, and they perhaps arrived in better condition and/or the staff had acquired the skills to manage complicated births and mortality dropped.

Similarly, in the government service area, comprehensive EmOC contributed to reduction in maternal mortality. Although deliveries in the facility was low in this area (13% in 2004) (1), a quarter of those who did use a facility were delivered by caesarean section (3% caesarean section, out of 11% skilled care during 2001-2005) (4). In a setting with such a low use of deliveries in the facility, it is likely that most caesarean cases were needed to save the life of the mother.

Skilled attendance in the government service area is equivalent to facility-use as there are no midwives in that area. However, midwives are available in the ICDDR,B service area for women residing there, providing a basic EmOC programme for normal births and referring women for comprehensive EmOC, if needed. While maternal mortality of those using the basic EmOC programme also experienced a decline over time (967 per 100,000 pregnancies during 1987-1991 to 267 per 100,000 pregnancies during 2002-2005), the rate of decline was not faster than that for women who did not seek skilled care at birth (207 per 100,000 pregnancies during 1987-1991 to 67 per 100,000 pregnancies during 2002-2005). Merely, the presence of ICDDR,B's basic EmOC services cannot effectively contribute to reduction in maternal mortality even with use as high as 40%.

These findings with regard to basic EmOC raises questions about the quality of care provided and of the referral system. This lack of effect could be partially explained by the fact that not all who were referred from the basic EmOC service providers accepted referral or accepted it immediately. Non-compliance was about 40-60% during 2003-2005 (Table 10).

**Table 10.** Percentageage of mothers referred for and admitted to a comprehensive EmOC facility outside the ICDDR,B service area

Year	No.	Referred (%)	No.	Admitted, facility outside ICDDR,B (%)
2003	3,214	7.5	240	40.0
2004	2,716	6.4	174	58.0
2005	2,644	7.5	197	57.9

Another explanation may be that more and more women with complications are now bypassing the ICDDR,B's basic EmOC programme and heading directly to the comprehensive EmOC facilities. Through qualitative research, the TBAs and village doctors stated that they have increased referrals over time directly to comprehensive EmOC facilities for cases that include the following complications: (a) baby's hand or leg came first; (b) the placenta was torn apart; (c) labour-pains did not increase even after administering injection (oxytocin) and saline; and (d) convulsions occurred just before birth of the baby. They also explained that referrals increased because of increased numbers of facilities, improved communications (including roads, telephones), and higher awareness among women and families. According to them, arranging money was and is still a barrier to accessing maternal service (e.g. comprehensive EmOC) for many families but that this barrier is overcome for life-threatening complications. Another motivating factor for increased referrals may be because the village doctors have developed relations with specific facilities where they refer complicated cases.

That those at home also have a decrease in maternal mortality also suggests that those with complications are now more aware of and deliver with a skilled attendant; more normal births make up the pool of those at home and in the Matlab basic EmOC programme. This is consistent with the fact that three-fourths (16 of 21) of all those who died during 2001-2005 came in contact with skilled attendance.

In both the areas, the causes of maternal death have shifted over time with substantial reductions in mortality due to haemorrhage, infection, and obstructed labour. The increased use of comprehensive EmOC and the easy availability of antibiotics most likely contributed to reductions in the number of death due to obstructed labour and infections (15). According to the findings of the qualitative study, the use of antibiotics by village doctors is very common; they stated during interviews that they prescribe antibiotics after delivery regardless of the status of the woman—normal or complicated. One village doctor with 40 years of experience reported prescribing antibiotics for complications, such as tears, fever, and, after delivery, for any sort of infection. Another village doctor said that the main reason for using antibiotics was to heal the birth-canal more quickly and to protect the mother from fever, pain, and infection. That they have been prescribing antibiotics over years was confirmed by an experienced (30 years) village doctor who said:

We have prescribed antibiotics from the day we started our private practice. We know that antibiotics prevent any sort of infection. Therefore, whenever I am called in for delivery in the home, I start prescribing antibiotics after delivery, whether the mother has any complication or not.

As blood is still not easily available, reduction in the number of deaths due to haemorrhage could be possible because the management of births improved, and harmful practices decreased. Despite these, haemorrhage remains the main killer in both the areas with eclampsia/pre-eclampsia being the second. Indirect causes of mortality, such as tuberculosis, hepatitis, and cardiovascular diseases, contribute to a larger proportion of indirect maternal deaths.

**Fig. 9.**
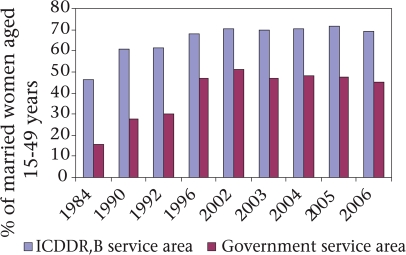
Contraceptive-use rate among married women in ICDDR,B surveillance sites, 1984-2005

The decline of fertility contributed to the reduction in maternal mortality in both ICDDR,B and government service areas but the effect was greater in the government area: 30% reduction in maternal mortality due to reduction in pregnancy rates in the government service area compared to 23% in the ICDDR,B service area over time. Reduction in lifetime risk for maternal death that takes into account both fertility effect and obstetric conditions was similar in both ICDDR,B (79%) and government (78%) service areas. Although the contraceptive prevalence rate (CPR) increased in both the areas during 1984-2005 (in the ICDDR,B service area from 46% during 1984 to 69% during 2006; and in the government service area from 16% in 1984 to 45% in 2006) (16), the ICDDR,B service area consistently maintained higher contraceptive prevalence rates compared to the government service area (Fig. 9). The effect of a relatively lower CPR on reduction in maternal mortality in the government service area was probably compensated for by a higher safe abortion rate in that area.

**Fig. 10.**
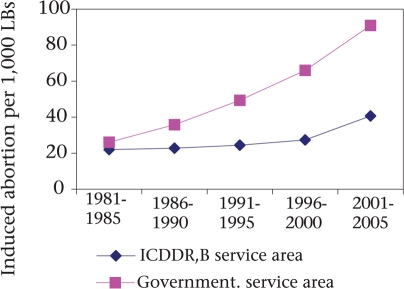
Abortion ratios among married women of reproductive age in ICDDR,B and government service areas, 1978-2005

Since 1979, access to safe menstrual regulation (MR) services has been available in the government and NGO facilities as the Government allowed MR to ensure the non-pregnant state (17). The abortion ratio, an indicator of the likelihood that a woman will abort a pregnancy if she becomes pregnant, increased in both the service areas during 1981-2005. However, the rate of increment in the government service area was much faster than that in the ICDDR,B service area (Fig. 10). In the early 1980s, the abortion ratio was below 20 per 1,000 livebirths in both the areas whereas, in 2001-2005, the ratio in the government service area (91 per 1,000 livebirths) was more than two times higher than the ICDDR,B service area ratio (40 per 1,000 livebirths).

LBs=Livebirths

Other factors that have likely impacted both the service areas include increased education of women and female literacy (26% in 1991 to 44% in 2003), women's empowerment efforts, and improved roads and communication (18). In recent years, Bangladesh has made significant progress, especially increasing access and gender equity in schooling at both primary and secondary levels. The female secondary education projects of the Government, initiated in the late 1990s, increased enrollment of girls in secondary schools from 32.6% during 1985 to 52.3% in 2005, according to the Bangladesh Bureau of Educational Information Statistics (19). Collateral-free credit programmes aimed at both alleviating poverty and increasing status of women are widespread in rural Bangladesh and have been shown to improve health outcomes, at least for children (20,21). The mechanism(s) by which these factors contribute to maternal mortality is/are not clear but maternal education is certainly a strong predictor of the use of services (5). The extensive development of roads and highways and increased numbers of vehicles and telecommunication over the past decade have also likely contributed to the reduced maternal mortality by making use of higher facility services for a safe delivery much easier.

In summary, access to and use of comprehensive EmOC services possibly is the major contributor to the reduction in maternal mortality in the ICDDR,B service area. In the government service area, although the use of comprehensive EmOC services contributed to reduction in maternal mortality, the effect was not as strong as that in the ICDDR,B service area. On the other hand, the effects of the TFR in the government service area had a larger effect on reduction in maternal mortality compared to the ICDDR,B service area. These are further confirmed by similar rates of reduction in both the areas for the lifetime risk of maternal mortality. Substantial reduction in abortion-related mortality in both the areas is explained by the family-planning programme of the Government that supports safe MR services. As a distal determinant, change is socioeconomic status, particularly education of women contributed to reduction in maternal mortality probably through the increased use of skilled care (5) in both the areas.

Even with this successful reduction in the maternal mortality, the current rate of reduction in maternal mortality in Bangladesh will achieve only about 75% of the MDG 5 target of 2015 (22). At the present rate of reduction, the maternal mortality ratio in Bangladesh will decline to about 190 per 100,000 livebirths by 2015. It must increase two-folds to achieve the MDG 5 target of 143 by 2015. Investment in further strengthening the comprehensive EmOC and the family-planning programme is clearly important and need to be pursued. Additional policies that bring expansion of female education, later childbearing, better financial access to the poor, and poverty alleviation are also essential to sustain the success achieved to date.

## ACKNOWLEDGEMENTS

The study was funded by the Department for International Development (DFID), UK through ICDDR,B (Grant No. 00335) and Research Program Consortium (RPC) Towards 4+5 to ICDDR,B (Grant No. 00458) by DFID, UK. The authors extend their thanks to the Health and Demographic Surveillance Unit of ICDDR,B for providing data for the study.
